# Heart Healthy Ohio Initiative: A Statewide Cooperative to Improve Cardiovascular Risk

**DOI:** 10.1007/s11606-026-10171-6

**Published:** 2026-01-26

**Authors:** Shari D. Bolen, Douglas Einstadter, Jordan Fiegl, Thomas E. Love, Jackson T. Wright, Aleece Caron, Eileen Seeholzer, Adam T. Perzynski, Chris Taylor, Leon McDougle, Stephanie Kanuch, Catherine Sullivan, Susan A. Flocke, Kurt C. Stange, Randy Wexler, Saundra Regan

**Affiliations:** 1 MetroHealth Population Health Research Institute, Cleveland, OH USA; 2 Case Western Reserve University (CWRU) School of Medicine, Cleveland, OH USA; 3 The Ohio State University Wexner Medical Center, Columbus, OH USA; 4 School of Medicine, Oregon Health and Science University, Portland, OR USA; 5 College of Medicine, University of Cincinnati, Cincinnati, OH USA

**Keywords:** primary care, quality improvement, hypertension, cardiovascular disease

## Abstract

**Background:**

Ohio ranks among the highest US states for cardiovascular disease (CVD) morbidity and mortality. Although interventions exist for managing CVD risk factors, adoption in primary care is often limited. The Agency for Healthcare Research and Quality funded four states to develop scalable, statewide models for implementing evidence-based practices to address these gaps.

**Objective:**

To evaluate the effectiveness of the Heart Healthy Ohio Initiative (HHOI), a statewide quality improvement (QI) initiative focused primarily on improving blood pressure (BP) control

**Design:**

Pre-post, repeated cross-sectional QI study using electronic health record (EHR) data to compare patient outcomes 6 months pre- and post-intervention

**Participants:**

A total of 293,638 adult patients (aged ≥ 18 years) from 48 primary care clinics across 21 counties, of whom 107,216 (37%) had hypertension.

**Interventions:**

Practices received structured QI support to implement evidence-based strategies for hypertension management, including BP measurement, timely follow-up, treatment protocols, and outreach. Monthly QI coaching, peer learning, and data feedback supported implementation. Smoking cessation strategies were also encouraged.

**Main Measures:**

The primary outcome was BP control, defined as < 140/90 mmHg. Process measures included repeat BP measurement, timely follow-up, and medication intensification. Regression analyses evaluated the impact of process measures on BP control improvement. Secondary measures on smoking cessation included screening, quit advice, resource referrals, and medications prescribed.

**Key Results:**

BP control improved from 67.7% to 70.7% post-intervention. Greater improvements were observed among rural and uninsured patients (> 6%) compared to smaller gains among Medicaid enrollees, younger patients, and women (~ 2%). All three process measures were significantly associated with better BP control in multivariable models. Smoking cessation measures were maintained or declined by ~ 2%, although only five sites actively addressed smoking cessation.

**Conclusions:**

HHOI demonstrates the feasibility and early success of a statewide, cooperative QI infrastructure to improve BP control. This model may be replicable in other states and offers insights for addressing hypertension control through targeted, scalable strategies.

**Supplementary Information:**

The online version contains supplementary material available at 10.1007/s11606-026-10171-6.

## BACKGROUND

Among US states, Ohio is in the highest quartile for cardiovascular (CVD) morbidity and mortality^1^ and has disparities in CVD risk factors by geographic region, race/ethnicity, and insurance type.^2–4^ Modifiable CVD risk factors such as hypertension, cholesterol, and smoking are important contributors to CVD morbidity, mortality, and health care cost.^5–7^ Many interventions have improved CVD risk factors in randomized trials,^8–14^ yet timely adoption of these evidence-based interventions in real-world primary care settings remains challenging.^15^

Quality improvement (QI) efforts within regional or multi-state cooperatives have been a successful model for accelerating evidence-based strategies from randomized trials into practice.^16–21^ The Agency for Healthcare Research and Quality (AHRQ) EvidenceNOW Advancing Heart Health initiative recently funded cross-state cooperatives to address cardiovascular risk factors in small independent and rural primary care practices showing improvements in QI capacity and CVD risk factors.^17,18,22–25^ Due to barriers when dealing with cross-state policies, AHRQ decided to fund pilots to establish statewide cardiovascular cooperatives designed to improve CVD care as part of an EvidenceNOW Building State Capacity initiative.^26^ Lessons learned from the statewide approach could then be used by other states in a future spread initiative on state-based healthcare extension cooperatives.^27^

In this report, we describe the implementation strategies and effectiveness of the Ohio statewide cardiovascular cooperative. We also provide detailed results for hypertension control, which was the main area of QI focus. Providing models for cooperative statewide QI efforts for cardiovascular health that engage payers and other partners could result in a greater ability for spread to other states to improve cardiovascular health across the USA.

## METHODS

### Study Design and Population

This article describes the formation of a statewide cooperative and evaluates the impact of a quality improvement project (QIP) on blood pressure (BP) control and smoking cessation using a repeated cross-sectional analysis of electronic health record (EHR) data from participating practices over time. We have followed the SQUIRE guidelines for reporting on QI interventions.^28^ For this report, we focus primarily on BP control since most practices focused their QI efforts in this area and it was our primary outcome. This study was determined not to be human subjects research by the MetroHealth Institutional Review Board (Protocol # 20–00885) and each University’s IRB separately. The study population consisted of 293,638 unique individuals 18 years of age or older and seeking care at one of 48 primary care practices participating in the QIP. Although originally conceived as a stepped wedge design with the QIP delivered in four waves each separated by 1 month, challenges in completing practice recruitment^29^ led us to re-design the analyses to reflect a pre-intervention vs. post-intervention design across all participating practices.

### Statewide Cooperative Infrastructure and Formation

The formation of the four statewide cooperatives has been described previously.^30^ In brief, the Heart Healthy Ohio Initiative (HHOI) included three academic medical centers located in the Northern (Case Western Reserve University), Central (The Ohio State University), and Southern (University of Cincinnati) regions of Ohio, which served as the backbone organizations for the cooperative, providing subject matter expertise, data and evaluation expertise, and clinical coaching. Three regional health improvement organizations in the Northern (Better Health Partnership), Central (Health Impact Ohio), and Southern (The Health Collaborative) regions of Ohio served as anchor organizations, assisting with recruitment, providing QI coaching to practices, and disseminating best practices. A Steering Committee of professional and QI organizations, payers, the Ohio Department of Health, the Ohio Department of Medicaid, the Ohio Association of Community Health Centers, and community partners met twice yearly to guide the project. The Ohio Academy of Family Physicians co-led the Dissemination, Engagement, and Sustainability Team and provided leadership in recruitment, dissemination, and sustainability, including offering maintenance of certification for clinicians. This infrastructure was built off a nascent cardiovascular cooperative called Cardi-OH, which funded the Ohio schools of medicine for dissemination of evidence-based strategies through the Medicaid Technical Assistance and Policy Program.^31^

We established the HHOI using a Collective Impact Model^32^ to bring all partners together. Table 1 outlines the infrastructure, roles, and activities of the cooperative partners. A virtual kickoff held in April 2021 included all partners (Steering Committee, project team, codesign practices, and patient representatives). The purpose of the kickoff was to discuss current activities to improve cardiovascular health around the state and to discuss the future vision of the cooperative. These facilitated discussions continued throughout the 6-month codesign period using biweekly virtual 1-h meetings with partners to finalize the HHOI infrastructure and codesign the Heart Healthy QIP intervention. During this period, we ratified the HHOI mission, developed a team-based care prototype, developed a change package (resources for practices), and key driver diagram (Appendix Fig. 1), determined the EHR data elements required for process and outcome measures, developed EHR data queries, and refined the implementation strategy. As part of codesign, from September 2021 through August 2022, we performed a 1-year pilot study of the QIP intervention and implementation strategies at 4 primary care practices and incorporated lessons learned from the pilot into the broader implementation.

**Table 1:** HHOI Infrastructure, Roles, and Activities

Organization	Role	Description of activities
Agency for Healthcare Research and Quality (AHRQ)	• Funder	• Managed TA partners and grantees for shared learning, dissemination, and timely deliverables
AHRQ-contracted Technical Assistance (TA)	• Centralized data and evaluation • Centralized facilitation and TA	• Standardized and analyzed selected data elements across state grantees • Facilitated shared learning across grantees • Provided TA related to barriers which arose for individual grantees
Steering Committee	• Guide vision and activities of the cooperative	• Helped recruit practices • Helped address barriers • Disseminate evidence-based best practices and shared learnings • Provided input on sustainability
Academic Medical Centers	• Academic expertise in cooperatives, codesign, cardiovascular health (CVH), and social drivers of health (SDoH) • EHR data extraction expertise • Primary care practice engagement and recruitment • Quality improvement (QI) coaching • Provided continuing medical education (CME) credits	• Developed change package with best practices for CVH and SDoH • Grew the cooperative and led the establishment of the QI support infrastructure • Led intervention codesign • Practice recruitment • Obtained legal agreements for practice data sharing • Developed of data elements and data dashboard • Provided clinical QI coaching • Evaluate and disseminate findings nationally • Provided CME
Regional Health Improvement Organizations	• Primary support for QI coaching • Practice recruitment and engagement • Dissemination of best practices	• Helped with practice recruitment • Led dissemination of findings to practices and partners • QI coaching
Ohio Academy of Family Practice	• Practice recruitment • Maintenance of certification (MOC) • Dissemination	• Helped with practice recruitment • Provided MOC credits • Co-led dissemination of findings to practices and partners
Primary care practices	• Conduct QI around hypertension care and smoking cessation by testing elements of the change package	• Tested the statewide QI infrastructure by piloting the QIP
Payers	• Reduce payer level barriers to CVH care	• Piloted ways for enrollees to obtain home blood pressure monitors • Sustained QI infrastructure

*Abbreviations*: *AHRQ*, Agency for Healthcare Research and Quality; *TA*, technical assistance; *SDoH*, social drivers of health; *CVH*, cardiovascular health; *CME*, continuing medical education; *MOC*, maintenance of certification; *QI*, quality improvement

### QIP Recruitment

We recruited 53 primary care practices from 18 health systems over a 1-year period using a warm hand-off approach.^33^ We attempted to recruit at least two practice champions (ideally one prescribing provider and one staff member) to lead activities at each site. Five practices dropped out due to capacity and time constraints (4 prior to beginning implementation and 1 after a few months), leaving 48 practices from 15 health systems.

### Interventions

We employed evidence-based strategies and focused on four key elements for hypertension management: (1) accurate BP measurement, including repeat BP measurement if the initial BP reading was elevated; (2) timely follow-up (monthly follow-up using staff-led visits that promoted treatment intensification to achieve BP control); (3) a treatment algorithm emphasizing once-daily low-cost medications; and (4) outreach to patients with elevated BP using an EHR-based registry. We also encouraged practices to identify and address health-related social needs, and to use communication resources for patient engagement. We provided all practices with a hypertension change package based on prior successful regional and statewide efforts for managing hypertension.^16,34–36^ We also employed evidence-based strategies focused on key elements for smoking cessation (i.e., screening, advising to quit, referring to smoking cessation resources such as the state quit line, and prescribing medications) and a toolkit for smoking cessation.^37^

### Implementation

Twenty-nine practices were invited to attend a virtual kickoff in April 2022, and the rest of the practices (*n* = 19) were invited to attend a virtual kickoff in June 2022 based on when sites were recruited. QI coaching began 1 to 2 months after each kickoff. We used the following implementation strategies: (1) QI coaching/practice facilitation; (2) Audit and feedback of EHR data; and (3) Peer-to-peer learning. At each virtual 3.5-h kickoff, we reviewed the change package for hypertension and smoking cessation, provided a high-level QI overview, and helped practices begin setting SMART (specific, measurable, achievable, relevant, and time-based) aims and planning for their Plan, Do, Study, Act cycles. QI coaches focused on BP strategies first unless a site was strongly interested in smoking cessation since BP control was our primary outcome. During the year following the kickoff, QI coaches met monthly with practices. Practice members were also invited to quarterly “Action Period” webinars that included a review of aggregate practice-level EHR data on processes and outcomes, peer-to-peer learning sessions, and presentations on clinical and/or QI topics. We also developed monthly podcasts on clinical and QI topics to support implementation efforts.

### Data Collection

After finalizing data use agreements, sites used secure file transfer protocols to submit EHR data monthly for all patients 18 years and older seen during the previous month to a central data processing center. The data included 50 variables describing demographics (age, sex, race/ethnicity, patient address), visit date and visit type, scheduled appointments, vital signs measured at each visit (all blood pressures and time measured, body mass index), ICD-10 codes for all diagnoses on the problem list, lab results (lipids, HbA1c), current medication information (medication name, start and stop dates, strength, dose, route), and information on tobacco use, advice to quit, and tobacco cessation referrals. We identified antihypertensive medications in the medication list using the Anti-Hypertensive Drugs Value Set.^38^ We counted the number of medication classes based on mechanism of action for each person at each visit. Combination medications were counted as multiple classes depending on their components. The medication classes used are shown in Appendix Table 1.

A unique patient identifier allowed longitudinal tracking of patients. After receipt, the data were transformed into a standard format and physiologically implausible values were removed. Site data were combined for aggregate reporting of study process and outcome measures on a web-based dashboard updated monthly with the new practice data. We also linked EHR data to the American Community Survey using zip code to obtain specific neighborhood-level socioeconomic data on household income, poverty, and high school graduation for cohort regression analyses.

### Process and Outcome Measures

Our primary outcome measure was the percent of adult patients with a diagnosis of hypertension and with their most recent BP controlled (< 140/90 mmHg). We used < 140/90 mmHg to match the national Healthcare Effectiveness Data and Information Set (HEDIS) quality measure^39^ although we promoted the individual goal of < 130/80 mmHg. Process measures for hypertension included (1) Repeat BP — the percent of adults with hypertension who had a documented repeat BP measurement at the same office visit if the first BP measurement was elevated (≥ 140/90 mmHg); (2) Timely Follow-up Scheduled — the percent of adults with hypertension and a final BP greater than 140/90 mmHg who received an appointment for an in-person or telehealth follow-up visit within 30 days; and (3) Number and type of antihypertensive medication classes for those with elevated BP (Appendix Table 1).

Secondary measures for smoking cessation included the percent of adults screened for smoking and, for smokers, the percent of adult smokers who received advice to quit, referral to smoking cessation resources, and prescribed medications to assist with quitting.

To evaluate disparities in process and outcome measures, we stratified by race/ethnicity (non-Hispanic Black, non-Hispanic White, Hispanic, and Other), and insurance type (Medicaid, Medicare, Commercial, and Self-pay).

### Data Analysis

For all blood pressure and smoking cessation measures, we compare the final visit measure in the 6-month post period to the final visit measure in the 6-month baseline period using cross-sectional EHR data reports from the two time periods using chi-squared tests for proportions and *t*-tests for continuous variables. For the primary hypertension outcome (BP control), we compared the final BP during the 6-month post-implementation period to the final BP in the 6 months pre-implementation using multivariate linear regression accounting for age, sex, insurance type, race, ethnicity, baseline systolic BP, and zip code-based socioeconomic status. To understand the effect of each of our three process measures (repeat BP, timely follow-up, and medication intensification) on the primary outcome of BP control using the final BP reading in the 6-month post-implementation period, we created cohorts for multivariate models for each process measure with covariate adjustment as described above. These cohorts required a patient to have been seen and have a BP reading in each of the three project phases (6 months pre-, 12 months during, and 6 months post-implementation). Additionally, the patient must have been in the denominator for the respective process measure during the 12-month implementation phase to be eligible. For instance, a patient had to have an elevated BP to be considered for a repeat BP or a scheduled follow-up within 1 month. We calculated antihypertension medication intensification using prescription information from the EHR. For individuals with an elevated BP (≥ 140/90 mmHg), we determined whether a new class of medication was ordered. Due to missing data, we were unable to include dose increases of the same medication or a change to a different medication in the same class in determining treatment intensification. Single imputation was used where patients had a biological sex categorized as “other” or race/ethnicity of “unknown.” Because each process measure was an encounter-based measure and patients could have multiple opportunities for process measures, the predictor was calculated as the proportion of opportunities met within the 12-month implementation period.

## RESULTS

### Study Population Characteristics

A total of 293,638 unique adult patients were seen at the 48 primary care practices that participated in the QIP, representing 15 health systems in 21 Ohio counties (Table 2). Of these, 37% (*n* = 107,216) had a diagnosis of hypertension and 23% were smokers (Table 2). The median age was 52 years and 56% were male. The distribution of race/ethnicity and insurance type were diverse with 23% non-Hispanic Black, 61% White, 5% Hispanic, and 12% Other/Unknown, and 17% Medicaid, 25% Medicare, 42% Commercial, and 14% Uninsured. Five percent were seen at a rural clinic. Neighborhood-level measures for this population showed that over 90% had graduated high school, 13% lived below the federal poverty line, and the median income was $59,321. These characteristics were similar for people with hypertension except for a higher median age of 62 years, a higher non-Hispanic Black population (30%), and a higher Medicare (39%) and lower Commercial (32%) population (Appendix Table 2).

**Table 2:** Study Population Characteristics for All Adults Seen at the 48 Practices

**Characteristic**	**Baseline** **(6 months)** ***N* = 182,694**^**1**^	**Implementation** **(1 year)** ***N* = 388,957**^**1**^	**Post-implementation** **(6 months)** ***N* = 203,069**^**1**^	**Overall unique patients** ***N* = 293,638**^**1**^
Race and ethnicity				
Non-Hispanic Black	47,144 (26%)	99,730 (26%)	51,034 (25%)	68,069 (23%)
Non-Hispanic White	109,696 (60%)	231,177 (59%)	122,417 (60%)	178,031 (61%)
Hispanic	8420 (4.6%)	17,772 (4.6%)	8911 (4.4%)	13,384 (4.6%)
Other	7662 (4.2%)	18,820 (4.8%)	9646 (4.8%)	14,107 (4.8%)
Unknown^2^	9772 (5.3%)	21,458 (5.5%)	11,061 (5.4%)	20,047 (6.8%)
Primary insurance				
Commercial	63,482 (35%)	148,456 (38%)	77,321 (38%)	122,975 (42%)
Medicaid	30,002 (16%)	70,359 (18%)	32,348 (16%)	48,562 (17%)
Medicare	61,500 (34%)	117,612 (30%)	60,873 (30%)	71,954 (25%)
Other	4820 (2.6%)	12,986 (3.3%)	3902 (1.9%)	7590 (2.6%)
Uninsured	22,890 (13%)	39,544 (10%)	28,625 (14%)	42,557 (14%)
Sex				
Female	78,139 (43%)	188,337 (48%)	89,280 (44%)	128,073 (44%)
Male	104,463 (57%)	200,343 (52%)	113,681 (56%)	165,368 (56%)
Other^2^	92 (< 0.1%)	277 (< 0.1%)	108 (< 0.1%)	197 (< 0.1%)
Age, years	58 (42, 70)	55 (39, 67)	56 (40, 68)	52 (36, 66)
Smoker	48,205 (26%)	93,210 (24%)	47,317 (23%)	68,691 (23%)
Hypertension	88,313 (48%)	185,669 (48%)	89,855 (44%)	107,216 (37%)
Seen at rural clinic	10,373 (5.7%)	22,604 (5.8%)	11,882 (5.9%)	13,737 (4.7%)
Neighborhood median income, $^3^	58,714 (44,157, 74,438)	59,746 (44,410, 76,926)	59,138 (44,494, 76,835)	59,321 (44,535, 77,129)
Unknown	157	497	3,345	3,169
Neighborhood % HS graduates^3^	91.3 (85.8, 94.6)	91.4 (85.8, 94.8)	91.4 (86.2, 94.8)	91.5 (86.7, 94.9)
Unknown	102	363	3,290	3,076
Neighborhood % living in poverty^3^	14 (7, 23)	13 (7, 22)	13 (7, 22)	13 (7, 22)
Unknown	126	429	3314	3122

^1^*n* (%); median (Q1, Q3); sample size differs due to the cross-sectional nature of the data and differing time periods

^2^Patients with “Unknown” race and ethnicity and/or with “Other” biological sex were recategorized if included in the modeling cohorts using single imputation to a more prevalent label for model interpretability purposes

^3^We linked electronic health record data to the American Community Survey using zip code to obtain the neighborhood-level measures of income, high school graduation, and poverty

### QI Focus Areas of Practices

Of the 48 practices, almost all focused QI efforts on blood pressure (*n* = 45). Of the 12 practices that had one QI coaching session for smoking cessation, two had no follow-up coaching related to smoking cessation, five began the smoking cessation QI activities near the end or after the 1-year implementation period due to initially focusing on BP control. This left five practices which actively worked on smoking cessation during the 1-year implementation (2 of which had a BP focus simultaneously).

### Measures Overall

Compared to the 6-month baseline period, the proportion of patients with BP control (< 140/90 mmHg) in the 6-month post-intervention period increased from 67.7% to 70.7% (Table 3). Among the unadjusted cross-sectional assessment of process measures (Table 3), repeat BP increased from 53.4% at baseline to 60.1% in the 6-month post-implementation period. Timely follow-up and medication intensification showed no overall improvements in the 6-month post- versus the 6-month pre-implementation period. The average number of BP medication classes went from 1.52 (SD 1.22) at baseline to 1.56 (SD 1.23) post-implementation.

**Table 3:** Unadjusted Blood Pressure and Smoking Cessation Measure Changes

Measure	Baseline	Post-intervention	Difference (% points)	95% CI of difference post vs. baseline
Blood pressure (baseline *n* = 62,695)				
BP < 140/90 mmHg	67.68%	70.69%	3.01	(2.50, 3.51)
BP < 130/80 mmHg	30.88%	34.46%	3.58	(3.07, 4.10)
BP repeated if elevated	53.37%	60.14%	6.77	(6.17, 7.37)
Timely follow-up	41.22%	36.49%	−4.73	(−5.50, −3.94)
Medication intensified	12.98%	12.52%	−0.46	(−1.04, 0.08)
Smoking cessation (baseline *n* = 134,622 for general population and 48,205 for active smokers)^1^				
Smoker	26.39%	23.30%	−3.09	(−3.36, −2.81)
Screened	92.40%	92.73%	0.33	(0.17, 0.50)
Advised to quit	63.43%	61.27%	−2.16	(−2.77, −1.55)
Referred to counseling	8.55%	6.56%	−1.99	(−2.32, −1.65)
Prescribed medication	21.52%	19.20%	−2.32	(−2.83, −1.81)

^1^General population sample size was used as the denominator for determining the percent of smokers and percent screened, while active smoker sample size was used as the denominator for advice to quit, referral to counseling and medications prescribed

Smoking rates also decreased from 26.4% to 23.3% (Table 3). However, smoking process measures remained the same or declined during the project. Screening for smoking remained high at about 92%. However, advice to quit, referral to resources, and medications prescribed had larger opportunities for improvement at baseline and all declined by about 2% (Table 3). Given the majority of clinics focused QI efforts on BP control (*n* = 45), we further report on BP control by subgroup and the association of hypertension process measure associations with BP control to better inform larger scale spread.

### Blood Pressure Control Measures by Subgroup

**Figure. 1:**
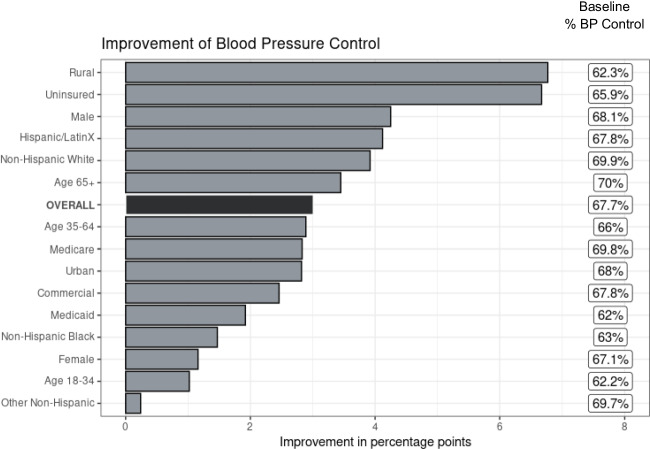
Improvement in blood pressure control overall and by subgroup.

**Figure. 2:**
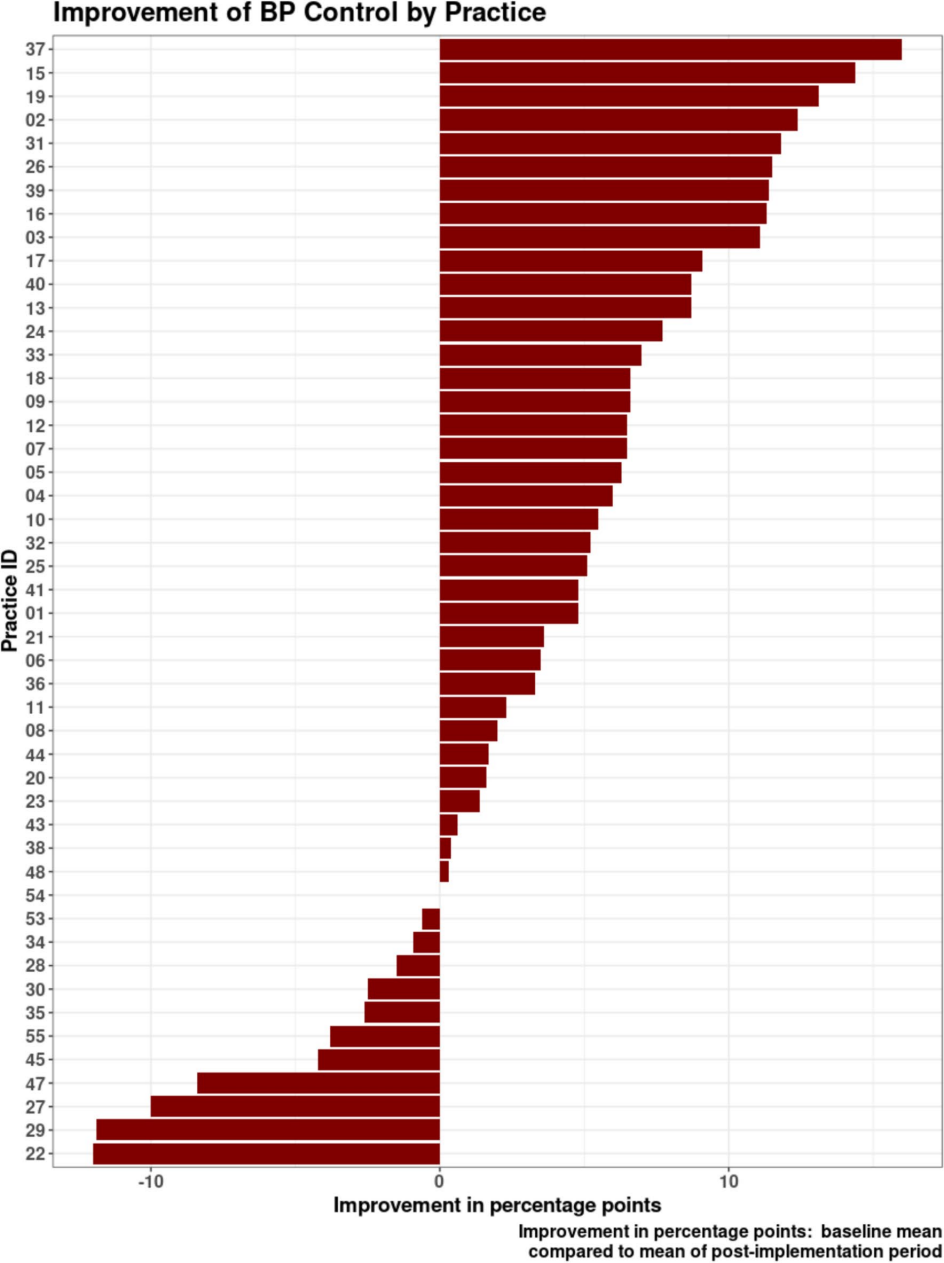
Unadjusted improvement in BP control by practice.

Increases in BP control differed by subgroup, with more than 6% improvement among rural and uninsured patients compared with a 2% improvement for Medicaid enrollees, women, and younger patients (Fig. 1, Appendix Table 3). Changes in BP control across practices were variable, ranging from a 3 or more percentage point increase (range in improvement 3.3% to 16.0%) in 28 practices to a 3 or more percentage point decrease (range, −3.8% to −12.0%) in 6 practices (Fig. 2).

### Processes Associated with Improvement in BP Control

**Figure. 3:**
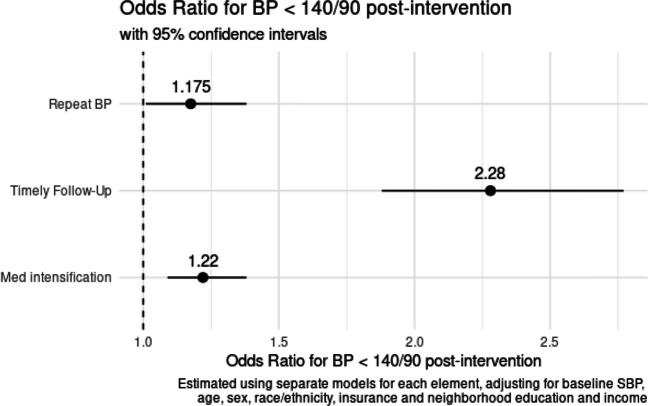
Association of process measures with post-intervention blood pressure control.

In the subsets of patients eligible for repeat BP, timely follow-up and/or medication intensification (Appendix Table 4 for study population characteristics), all three process measures were strongly associated with post-intervention improvements in BP control after adjusting for patient demographics (Fig. 3, Appendix Tables 5–7).

## DISCUSSION

HHOI is one of the first large-scale efforts to establish a statewide QI infrastructure for primary care practices. Through close collaboration of three colleges of medicine, three regional health improvement organizations, and numerous partners, HHOI was able to recruit and support primary care teams statewide in efforts to improve BP control. Practices achieved an overall 3 percentage point improvement in BP control during the 1-year implementation period. Our results demonstrated variability across subgroups, with greater improvements in BP control seen for rural and uninsured patients and lesser improvements for women, younger ages, and Medicaid enrollees. Outcomes by practice were also quite variable, with 28 practices showing a greater than 3 percentage point improvement (range in improvement 3.3% to 16.0%) and 6 practices showing a 3 or more percentage point worsening of BP control (−3.8% to −12.0%). Among process measures, obtaining a repeat BP was the sole measure to show an increase post-implementation for all practices combined. However, we found that all three process measures (repeat BP, timely follow-up, and medication intensification) were associated with increased post-implementation BP control in cohort analyses, suggesting the important role of these processes in achieving BP control. While the smoking rate declined by 3% pre- to post-implementation, process measures such as screening, advice to quit, referral to resources, and prescribing medications remained the same or declined slightly. However, few practices focused on smoking cessation efforts during the 1-year implementation period.

Previous multi-state or regional cooperatives have shown improvements in BP control using similar evidence-based interventions along with QI facilitation.^16–21^ Of note, the AHRQ EvidenceNOW Advancing Heart Health Initiative included seven regional cooperatives and more than 1500 small to medium sized primary care practices in 11 states.^23,25^ Practices within all seven cooperatives showed increased QI capacity and improved CVD risk factors (including a 7.3% increase in smoking cessation and a 1.6% increase in BP control).^25^ In 2021, AHRQ launched EvidenceNOW: Building State Capacity to catalyze the creation or enhancement of such an infrastructure in four States including Ohio.^26^ These statewide models including HHOI can provide useful lessons learned for other states that may wish to implement similar programs.

Our study adds to the literature by describing the infrastructure of a statewide model for CVD improvement which supports QI within diverse primary care practices. We also demonstrate our ability to use the infrastructure to improve BP control overall and within subgroups. Regular QI coaching, quarterly webinars with peer-to-peer learning and expert didactics, presenting monthly EHR data on process and outcome measures to support audit and feedback and to build competition, and building strategic partnerships at the state level were key aspects of success for our state cooperative. Further, while prior literature has shown the impact of repeat BP, timely follow-up, and medication intensification on BP control within a single health system,^35,40,41^ this study extends those findings by evaluating which processes contribute to BP improvements within QI interventions after adjusting for other variables known to impact BP control.

Our study had several limitations. First, we used a pre-post, repeated cross-sectional analysis for measuring change in BP control and had no fixed comparison group. Thus, we are unable to control for background temporal changes and do not know whether BP control would have improved by the same amount for a true control group. However, a recent national study demonstrated a worsening of BP control over time^42^ suggesting that our intervention had a positive effect. Second, we are unable to account for unobserved variables which may have led to some residual confounding. Third, some sites had difficulty extracting EHR data to allow calculation of medication intensification and timely follow-up. This likely decreased the power to identify any changes in these measures and may have biased the results. Finally, the result of BP improvement could have been primarily through accurate BP measurement. Our cohort analyses suggest other areas such as timely follow-up and medication intensification also influenced BP control; however, greater focus on these other measures in future QI efforts will be necessary to impact CVD morbidity and mortality.

Despite these potential weaknesses, the strengths of this study were its large sample size, which improves the precision of our outcome estimates; inclusion of multiple types of clinics across Ohio, which improves generalizability; and partner engagement, which assisted us with sustainability. We met routinely with the Ohio Department of Medicaid throughout the 3-year project for input. Near project end, they funded all 7 medical schools across the state along with a centralized coordinating body to establish regional QI Hubs through the Medicaid Technical Assistance and Policy Program. The goal was to better reach Medicaid enrollees and realize statewide population health improvements. ^43,44^ This has allowed Ohio to sustain a modified statewide infrastructure for QI support to primary care practices focused on diabetes and hypertension after completion of HHOI and which tailors to diverse regions of the state.

In summary, we report on one of the first statewide models for cardiovascular health improvements which combined the resources of three medical schools, three regional health improvement organizations, and multiple strategic partners across the state of Ohio to support and later sustain a QI infrastructure to successfully implement evidence-based best practices for CVD risk factor improvement in primary care. Investments in similar statewide approaches have the potential to result in large-scale health improvements. Further attention to ways coaches can support practices in improving medication intensification and timely follow-up would likely yield larger improvements. While all patient subgroups experienced improvement in BP control, some subgroups had larger improvements than others, emphasizing the need for future tailored efforts within targeted populations including younger ages, Medicaid enrollees, and non-Hispanic Blacks. Lastly, consideration of how to support sites to focus on more than one QI topic area such as blood pressure and smoking cessation simultaneously or ensuring more than 1 year to allow practices to conduct sequential QI projects will be needed when thinking about future initiatives.

## Supplementary Information

Below is the link to the electronic supplementary material.

ESM 1 (DOCX 116 KB)

## Data Availability

Data are not publicly available due to restrictions in data use agreements from participating partners sites.
