# Supplementary material for: Heart Healthy Ohio Initiative: A Statewide Cooperative to Improve Cardiovascular Risk
Source: J Gen Intern Med. 2026 Jan 26;41(7):1909–19. doi: 10.1007/s11606-026-10171-6 (PMC13176369; doi:10.1007/s11606-026-10171-6)
Supplement: Supplementary file 1 — (DOCX 116 KB) [file 11606_2026_10171_MOESM1_ESM.docx]

**Appendix**

**Appendix Figure 1: Key Driver Diagram**


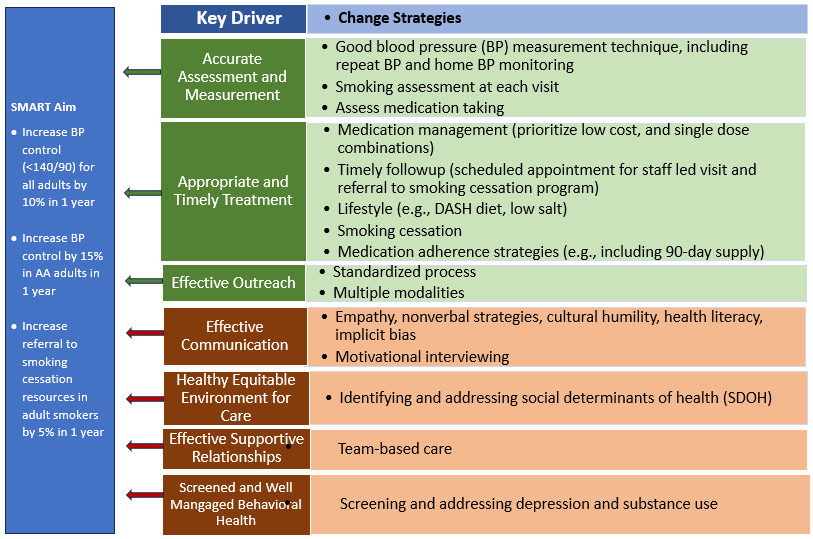


**Appendix Table 1: Anti-hypertensive drug classes used for number and type of medication classes for continuous quality improvement**

1. Loop diuretics
2. Thiazide or thiazide like diuretics
3. Beta-blockers
4. Angiotension converting enzyme-inhibitors
5. Angiotensin receptor blockers
6. Calcium channel blockers
7. Alpha-1 blockers
8. Alpha-2 agonists
9. Centrally acting agents
10. Peripheral adrenergic inhibitors
11. Vasodilators
12. Renin angiotensin system inhibitors

***We also showed practices doses of chlorthalidone, hydrochlotohiazide and amlodipine to try and promote higher doses of amlodipine and thiazide diuretics (higher dose of hydrochlorothiazide of 25 or 50 mg instead of 12.5 mg or switch from hydrochlorothiazide of 12.5 or 25 mg to chlorthalidone 25 mg to promote higher doses of diuretics and longer acting blood pressure medications which may be more forgiving of missed doses).

| **Appendix Table 2: Characteristics for Patients with Hypertension** | | | | |
| --- | --- | --- | --- | --- |
| **Characteristic** | **Baseline**  **period**  **(6-month)**  N = 62,695*^1^* | **Implementation**  **period**  **(1-year)**  N = 89,183*^1^* | **Post –** **Implementation**  **(6-month)**  N = 64,371*^1^* | **Overall unique patients**  N = 107,216 |
| Race and Ethnicity |  |  |  |  |
| Non-Hispanic Black | 18,454 (29%) | 25,870 (29%) | 19,155 (30%) | 31,739 (30%) |
| Non-Hispanic White | 36,362 (58%) | 51,374 (58%) | 36,947 (57%) | 60,913 (57%) |
| Hispanic | 2,607 (4.2%) | 3,567 (4.0%) | 2,638 (4.1%) | 4,419 (4.1%) |
| Other | 2,357 (3.8%) | 3,327 (3.7%) | 2,440 (3.8%) | 4,100 (3.8%) |
| Unknown^2^ | 2,915 (4.6%) | 5,045 (5.7%) | 3,191 (5.0%) | 6,045 (5.6%) |
| Primary Insurance |  |  |  |  |
| Commercial | 18,887 (30%) | 28,150 (32%) | 20,002 (31%) | 34,307 (32%) |
| Medicaid | 8,526 (14%) | 12,272 (14%) | 8,445 (13%) | 15,690 (15%) |
| Medicare | 27,244 (43%) | 35,541 (40%) | 26,309 (41%) | 41,377 (39%) |
| Other | 1,832 (2.9%) | 2,763 (3.1%) | 1,382 (2.1%) | 3,229 (3.0%) |
| Uninsured | 6,206 (9.9%) | 10,457 (12%) | 8,233 (13%) | 12,613 (12%) |
| Sex |  |  |  |  |
| Female | 26,020 (42%) | 36,696 (41%) | 26,094 (41%) | 44,815 (42%) |
| Male | 36,663 (58%) | 52,468 (59%) | 38,264 (59%) | 62,378 (58%) |
| Other^2^ | 12 (<0.1%) | 19 (<0.1%) | 13 (<0.1%) | 23 (<0.1%) |
| Age, years | 63 (53, 72) | 62 (52, 72) | 63 (53, 72) | 62 (51, 71) |
| Seen at rural clinic | 3,027 (4.8%) | 4,157 (4.7%) | 3,281 (5.1%) | 4,689 (4.4%) |
| Neighborhood Median Income, $^3^ | 58,980 (43,750, 74,759) | 59,250 (44,157, 75,035) | 58,714 (44,129, 75,667) | 58,980 (43,750, 74,940) |
| Unknown | 43 | 133 | 730 | 795 |
| Neighborhood HS Graduates, %^3^ | 91.2 (85.8, 94.6) | 91.3 (85.9, 94.6) | 91.2 (85.9, 94.7) | 91.3 (85.8, 94.6) |
| Unknown | 21 | 97 | 711 | 747 |
| Neighborhood below Federal Poverty Line, %^3^ | 13 (7, 23) | 13 (7, 23) | 14 (7, 23) | 13 (7, 23) |
| Unknown | 34 | 122 | 721 | 778 |

*^1^*n (%); Median (Q1, Q3); sample size differs due to the cross-sectional nature of the data and differing time periods.

^2^ Patients with “Unknown” race and ethnicity and/or with “Other” biological sex were recategorized if included in the modeling cohorts using single imputation to a more prevalent label for model interpretability purposes.

^3^ We linked electronic health record data to the American Community Survey using zip code to obtain the neighborhood-level measures of income, high school graduation and poverty.

**Appendix Table 3: Blood Pressure Control Improvement Overall and by Subgroup**

|  | Baseline | | Post-Intervention | | Difference | 95% CI of difference post vs base |
| --- | --- | --- | --- | --- | --- | --- |
|  | Subjects | BP < 140/90 | Subjects | BP < 140/90 | (% points) |  |
| Overall | 63,445 | 67.68% | 65,084 | 70.69% | 3.01 | (2.30, 3.32) |
| Age (years)  18-34  35-64  65+ | 2,487 (4%)  31,826 (50%)  29,132 (46%) | 62.16%  66.02%  69.98% | 2,686 (4%)  33,355 (51%)  29,043 (45%) | 63.18%  68.90%  73.43% | 1.02  2.88  3.45 | (-1.66, 3.69)  (2.16, 3.61)  (2.72, 4.19) |
| Sex^1^  Female  Male | 26,410 (42%)  37,022 (58%) | 67.14%  68.07% | 26,454 (41%)  38,617 (59%) | 68.30%  72.32% | 1.16  4.25 | (0.36, 1.96)  (3.60, 4.90) |
| Insurance  Medicare  Commercial  Medicaid  Uninsured | 27,867 (44%)  20,428 (32%)  8,861 (14%)  6,289 (10%) | 69.77%  67.84%  62.00%  65.94% | 26,857 (41%)  21,168 (33%)  8,745 (13%)  8,314 (13%) | 72.60%  70.30%  63.92%  72.61% | 2.83  2.46  1.92  6.67 | (2.07, 3.59)  (1.57, 3.35)  (0.48, 3.36)  (5.15, 8.20) |
| Race/Ethnicity  Non-Hisp. Black  Non-Hisp. White  Hispanic/Latinx  Other non-Hisp. | 19,357 (31%)  38,853 (61%)  2,823 (4%)  2,412 (4%) | 63.03%  69.87%  67.80%  69.73% | 20,118 (31%)  39,607 (61%)  2,831 (4%)  2,528 (4%) | 64.50%  73.79%  71.92%  69.98% | 1.47  3.92  4.12  0.25 | (0.47, 2.38)  (3.29, 4.55)  (1.73, 6.58)  (-2.32, 2.88) |
| Practice Location  Urban  Rural | 60,333 (95%)  3,112 (5%) | 67.96%  62.28% | 61,653 (95%)  3,431 (5%) | 70.78%  69.05% | 2.82  6.77 | (2.30, 3.33)  (4.44, 9.10) |

There are 13 subjects at baseline and 13 at post-intervention whose sex is listed as “Other”.

Abbreviations: Non-Hisp. = Non Hispanic; BP = blood pressure; CI = confidence interval

**Appendix Table 4: Study Population Characteristics for Model Cohorts**

| **Characteristic** | **Repeat BP**  N = 19,669*^1^* | **Timely Follow-up and Med Intensification**  N = 15,956*^1^* |
| --- | --- | --- |
| Race-Ethnicity |  |  |
| Non-Hispanic White | 11,215 (57%) | 8,772 (55%) |
| Non-Hispanic Black | 6,239 (32%) | 5,387 (34%) |
| Non-Hispanic Other | 601 (3.1%) | 525 (3.3%) |
| Hispanic | 784 (4.0%) | 639 (4.0%) |
| Unknown | 830 (4.2%) | 633 (4.0%) |
| Primary Insurance |  |  |
| Commercial | 5,334 (27%) | 4,324 (27%) |
| Medicaid | 2,177 (11%) | 1,964 (12%) |
| Medicare | 9,611 (49%) | 7,835 (49%) |
| Other | 244 (1.2%) | 239 (1.5%) |
| Uninsured | 2,303 (12%) | 1,594 (10.0%) |
| Sex |  |  |
| Female | 7,886 (40%) | 7,027 (44%) |
| Male | 11,781 (60%) | 8,928 (56%) |
| Other | 2 (<0.1%) | 1 (<0.1%) |
| Age | 66 (56, 74) | 65 (56, 73) |
| Neighborhood  Median Income | 57,861 (42,890, 73,197) | 57,732 (41,784, 73,219) |
| Unknown | 18 | 15 |
| Neighborhood % HS Graduates | 90.7 (85.8, 94.4) | 90.6 (85.6, 94.4) |
| Unknown | 13 | 11 |
| Neighborhood % Living in Poverty | 14 (7, 23) | 15 (8, 24) |
| Unknown | 15 | 12 |
| *^1^*n (%); Median (Q1, Q3)  Abbreviations: BP = blood pressure; HS = high school | | |

**Appendix Table 5: Repeat Blood Pressure Measurement Association with Post-Intervention Blood Pressure Control**

| Term | Odds  Ratio | Standard Error | 95% CI (Lower) | 95% CI (Upper) | P-value |
| --- | --- | --- | --- | --- | --- |
| Repeated BP if BP elevated | 1.175 | 0.080 | 1.005 | 1.375 | 0.043 |
| Age (in 10 years) | 0.930 | 0.014 | 0.904 | 0.957 | <0.001 |
| Sex |  |  |  |  |  |
| Female | — | — | — | — | — |
| Male | 0.894 | 0.033 | 0.839 | 0.953 | 0.001 |
| Insurance Financial Class |  |  |  |  |  |
| Commercial / Private | — | — | — | — | — |
| Medicaid | 0.972 | 0.055 | 0.872 | 1.084 | 0.613 |
| Medicare | 1.058 | 0.041 | 0.977 | 1.146 | 0.163 |
| Uninsured | 0.802 | 0.053 | 0.723 | 0.889 | <0.001 |
| Race/Ethnicity |  |  |  |  |  |
| Non-Hispanic White | — | — | — | — | — |
| Non-Hispanic Black | 0.881 | 0.037 | 0.819 | 0.947 | 0.001 |
| Non-Hispanic Other | 0.897 | 0.085 | 0.760 | 1.061 | 0.203 |
| Hispanic | 1.193 | 0.080 | 1.020 | 1.397 | 0.028 |
| Median Income (in $1000) | 1.002 | 0.001 | 0.999 | 1.004 | 0.199 |
| High School Graduation | 1.001 | 0.004 | 0.993 | 1.009 | 0.836 |
| Poverty (below federal poverty limit) | 1.001 | 0.003 | 0.996 | 1.006 | 0.722 |
| Baseline Systolic Blood Pressure | 0.976 | 0.001 | 0.975 | 0.978 | <0.001 |

Abbreviations: BP = blood pressure, CI = confidence interval

Appendix Table 5 describes odds ratio estimates from a logistic regression model for post-intervention BP control (< 140/90) as a function of Repeat Blood Pressure Adherence: defined as the proportion of opportunities in the post-intervention follow-up period where a repeat blood pressure was taken (so that 0 = never and 1 = each time.) The model adjusts for the following covariates: age (shown here in 10 year increments), sex as Male vs. not Male, primary insurance, race-ethnicity, neighborhood socio-economic status developed from ZCTA codes to represent home address for the subjects, including median income in thousands of dollars, the percentages of High School graduates among residents over the age of 25, and the percentage of residents living below the federal poverty line) as well as the final recorded systolic blood pressure value in the baseline period. Unknown values of primary insurance and of race-ethnicity were singly imputed using chained equations to mirror our known distribution within participating practices. Exponentiated coefficients are shown, along with standard errors and p values, accompanied by 95% confidence intervals. The intercept term is included in the model but omitted from Appendix Table 5.

**Appendix Table 6: Timely Follow-up Association with Post-Intervention Blood Pressure Control**

| Term | Odds  Ratio | Standard Error | 95% CI (Low) | 95% CI (High) | P-value |
| --- | --- | --- | --- | --- | --- |
| Timely follow-up if BP elevated | 2.281 | 0.098 | 1.883 | 2.768 | 0.000 |
| Age (in 10 years) | 0.927 | 0.016 | 0.899 | 0.955 | 0.000 |
| Sex |  |  |  |  |  |
| Female | — | — | — | — | — |
| Male | 0.906 | 0.035 | 0.846 | 0.970 | 0.005 |
| Insurance Financial Class |  |  |  |  |  |
| Commercial / Private | — | — | — | — | — |
| Medicaid | 0.985 | 0.059 | 0.879 | 1.106 | 0.802 |
| Medicare | 1.082 | 0.045 | 0.990 | 1.182 | 0.083 |
| Uninsured | 0.784 | 0.062 | 0.695 | 0.885 | 0.000 |
| Race/Ethnicity |  |  |  |  |  |
| Non-Hispanic White | — | — | — | — | — |
| Non-Hispanic Black | 0.913 | 0.040 | 0.843 | 0.988 | 0.023 |
| Non-Hispanic Other | 0.898 | 0.091 | 0.752 | 1.075 | 0.239 |
| Hispanic | 1.249 | 0.088 | 1.053 | 1.486 | 0.011 |
| Median Income (in $1000) | 1.003 | 0.001 | 1.000 | 1.005 | 0.041 |
| High School Graduation | 1.000 | 0.005 | 0.991 | 1.009 | 0.957 |
| Poverty (below federal poverty limit) | 1.000 | 0.003 | 0.994 | 1.006 | 0.915 |
| Baseline Systolic Blood Pressure | 0.977 | 0.001 | 0.975 | 0.979 | 0.000 |

Abbreviations: BP = Blood pressure; CI = confidence interval

Appendix Table 6 describes odds ratio estimates from a logistic regression model for post-intervention BP control (< 140/90) as a function of Timely Follow-Up Adherence: defined as the proportion of opportunities in the post-intervention follow-up period where timely follow-up was completed (so that 0 = never and 1 = each time.) The model adjusts for the following covariates: age (shown here in 10 year increments), sex as Male vs. not Male, primary insurance, race-ethnicity, neighborhood socio-economic status developed from ZCTA codes to represent home address for the subjects, including median income in thousands of dollars, the percentages of High School graduates among residents over the age of 25, and the percentage of residents living below the federal poverty line) as well as the final recorded systolic blood pressure value in the baseline period. Unknown values of primary insurance and of race-ethnicity were singly imputed using chained equations to mirror our known distribution within participating practices. Exponentiated coefficients are shown, along with standard errors and p values, accompanied by 95% confidence intervals. The intercept term is included in the model but omitted from Appendix Table 6.

**Appendix Table 7: Medication Intensification Association with Post-Intervention Blood Pressure Control**

| Term | Odds  Ratio | Standard Error | 95% CI (Low) | 95% CI (High) | P-value |
| --- | --- | --- | --- | --- | --- |
| Medication intensified if BP elevated | 1.224 | 0.061 | 1.087 | 1.379 | 0.001 |
| Age (in 10 years) | 0.932 | 0.016 | 0.904 | 0.961 | 0.000 |
| Sex |  |  |  |  |  |
| Female | — | — | — | — | — |
| Male | 0.925 | 0.035 | 0.864 | 0.990 | 0.025 |
| Insurance Financial Class |  |  |  |  |  |
| Commercial / Private | — | — | — | — | — |
| Medicaid | 0.976 | 0.059 | 0.870 | 1.095 | 0.676 |
| Medicare | 1.073 | 0.045 | 0.982 | 1.172 | 0.121 |
| Uninsured | 0.818 | 0.062 | 0.725 | 0.923 | 0.001 |
| Race/Ethnicity |  |  |  |  |  |
| Non-Hispanic White | — | — | — | — | — |
| Non-Hispanic Black | 0.897 | 0.040 | 0.829 | 0.971 | 0.007 |
| Non-Hispanic Other | 0.888 | 0.091 | 0.743 | 1.063 | 0.193 |
| Hispanic | 1.251 | 0.088 | 1.054 | 1.488 | 0.011 |
| Median Income (in $1000) | 1.002 | 0.001 | 0.999 | 1.004 | 0.184 |
| High School Graduation | 1.001 | 0.005 | 0.992 | 1.010 | 0.847 |
| Poverty (below federal poverty limit) | 1.000 | 0.003 | 0.994 | 1.005 | 0.877 |
| Baseline Systolic Blood Pressure | 0.977 | 0.001 | 0.975 | 0.979 | 0.000 |

Abbreviations: BP = Blood pressure; CI = confidence interval

Appendix Table 7 describes odds ratio estimates from a logistic regression model for post-intervention BP control (< 140/90) as a function of medication intensification: defined as the proportion of opportunities in the post-intervention follow-up period where medication intensification occurred (so that 0 = never and 1 = each time.) The model adjusts for the following covariates: age (shown here in 10 year increments), sex as Male vs. not Male, primary insurance, race-ethnicity, neighborhood socio-economic status developed from ZCTA codes to represent home address for the subjects, including median income in thousands of dollars, the percentages of High School graduates among residents over the age of 25, and the percentage of residents living below the federal poverty line) as well as the final recorded systolic blood pressure value in the baseline period. Unknown values of primary insurance and of race-ethnicity were singly imputed using chained equations to mirror our known distribution within participating practices. Exponentiated coefficients are shown, along with standard errors and p values, accompanied by 95% confidence intervals. The intercept term is included in the model but omitted from Appendix Table 7.

**Appendix Table 8: Heart Healthy Ohio Initiative Team Members and Affiliations**

| **Team member** | **Affiliation** |
| --- | --- |
| Shari D. Bolen MD, MPH | MetroHealth Population Health Research Institute, Case Western Reserve University (CWRU) School of Medicine |
| Aleece Caron, PhD | MetroHealth Population Health Research Institute, CWRU School of Medicine |
| Douglas Einstadter MD, MPH | MetroHealth Population Health Research Institute, CWRU School of Medicine |
| Thomas E. Love, PhD | MetroHealth Population Health Research Institute, CWRU School of Medicine |
| Adam T. Perzynski, PhD | MetroHealth Population Health Research Institute, CWRU School of Medicine |
| Eileen Seeholzer, MD, MS | MetroHealth Population Health Research Institute, CWRU School of Medicine |
| Jordan Fiegl, MS | MetroHealth Population Health Research Institute |
| Hannah Hill, MS | MetroHealth Population Health Research Institute |
| Stephanie Kanuch, MED | MetroHealth Population Health Research Institute |
| Catherine Sullivan, RD, MS | MetroHealth Population Health Research Institute |
| Kurt Stange, MD, PhD | CWRU School of Medicine |
| Richard Buchanon, PhD | CWRU Weatherhead School of Management |
| Manpreet Kaur, MA | CWRU, College of Arts and Sciences |
| Chris Bernheisel, MD | University of Cincinnati, College of Medicine |
| Jackie Knapke, MEd, PhD | University of Cincinnati, College of Medicine |
| Saundra Regan, PhD | University of Cincinnati, College of Medicine |
| Mary Beth B. Vonder Meulen, RN | University of Cincinnati, College of Medicine |
| Leon McDougle, MD, MPH | The Ohio State University Wexner Medical Center, Family Medicine |
| Chris Taylor, RD, PhD | The Ohio State University Wexner Medical Center, School of Health and Rehabilitation Sciences |
| Randy Wexler, MD, MPH | The Ohio State University Wexner Medical Center, Family Medicine |
| Deborah Cohen, PhD | Oregon Health & Science University, School of Medicine |
| Susan Flocke, PhD | Oregon Health & Science University, School of Medicine |
| Carrie Baker, CEO | Health Impact Ohio |
| Caroline Carter, LSW, MS | Health Impact Ohio |
| Brittany Daniels, MS | Health Impact Ohio |
| Amanda Sebastion, BSN, MSN | The Health Collaborative |
| Marty Williams, MSW, MA | The Health Collaborative |
| Tiffany Mattingly, MSN, RN | The Health Collaborative |
| Kate Mahler, CAE | Ohio Association of Family Physicians |
| Rita Horwitz, RN, CEO | Better Health Partnership |
